# Supplementary material for: Young Shoots of Red Beet and the Root at Full Maturity Inhibit Proliferation and Induce Apoptosis in Breast Cancer Cell Lines
Source: Int J Mol Sci. 2023 Apr 7;24(8):6889. doi: 10.3390/ijms24086889 (PMC10138517; doi:10.3390/ijms24086889)
Supplement: Supplementary file 1 [file ijms-24-06889-s001.zip › ijms-2282842-supplementary.pdf]

(a)

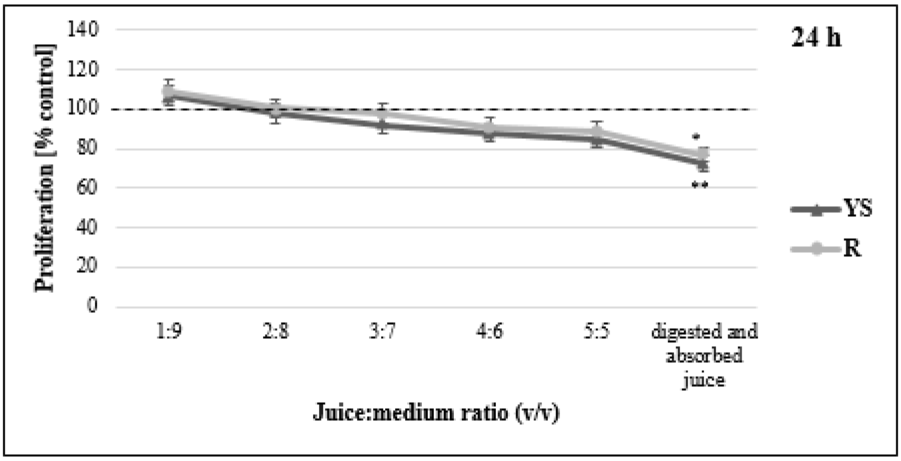

(b)

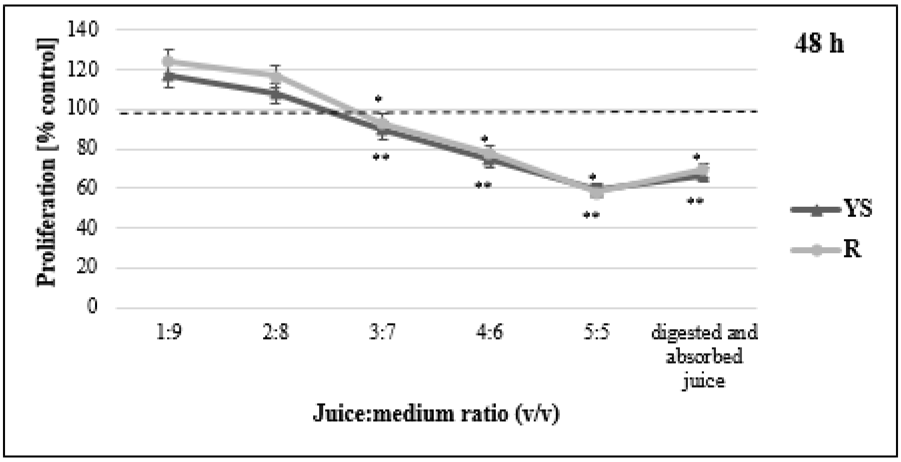

(c)

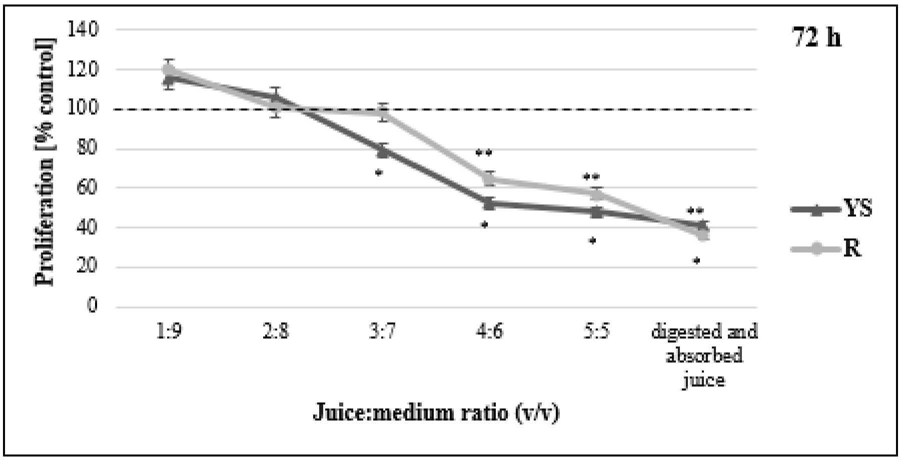

Supplementary Figure S1. Changes in the proliferation of MCF-7 cancer cells line at 24 (**a**), 48 (**b**) and 72 (**c**) hours.

YS - native juice of young shoots of beetroot, R - native juice of beetroot

* - statistically significant differences for the proliferation value at the YS point and at the point for the juice after digestion and absorption in relation to the control

** - statistically significant differences for the proliferation value at point R and at the point for the juice after digestion and absorption in relation to the control

$p \leq 0,05$; the Mann-Whitney U test

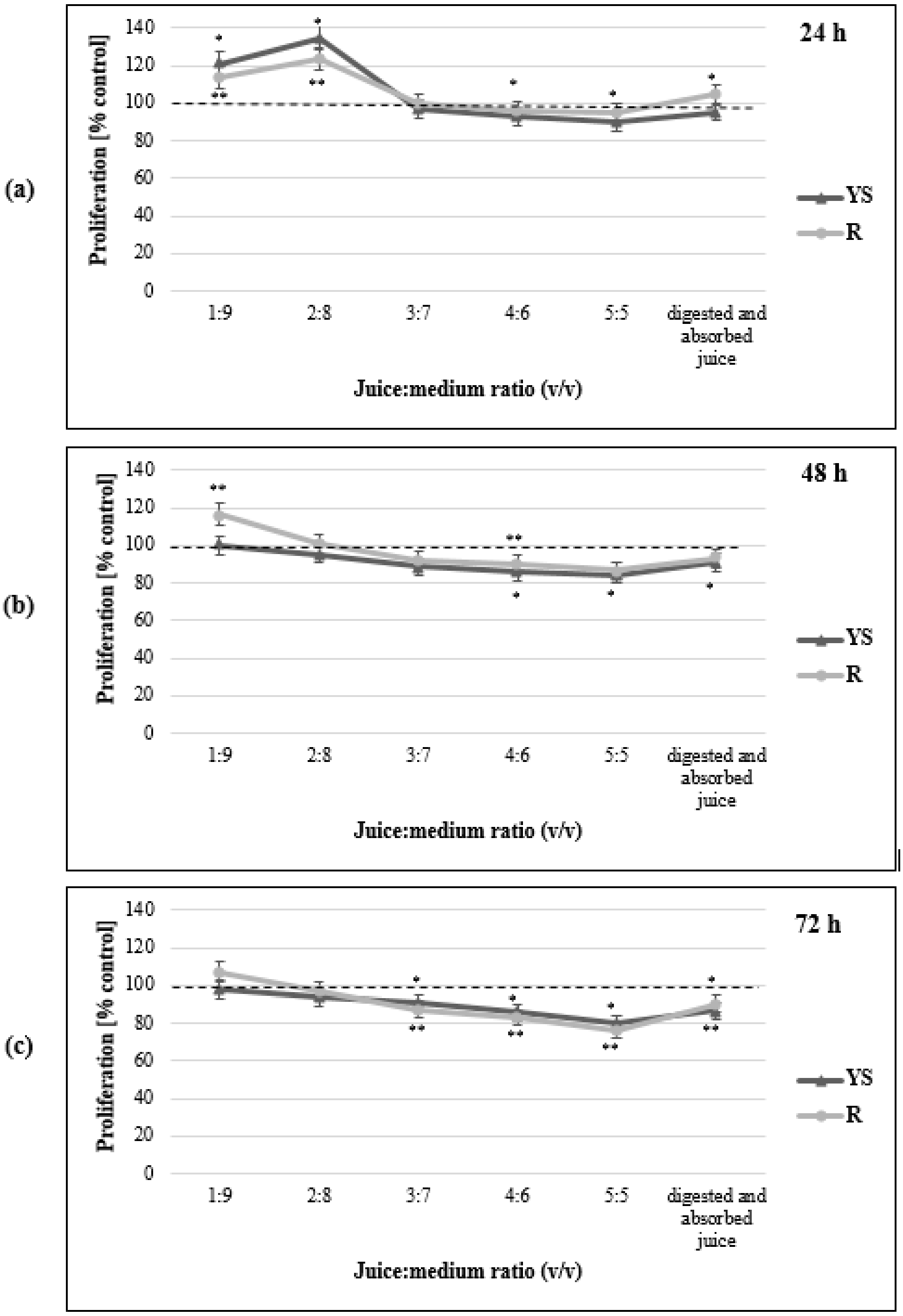

Supplementary Figure S2. Changes in the proliferation of MDA-MB-231 cancer cells line at 24 (**a**), 48 (**b**) and 72 (**c**) hours.

YS - native juice of young shoots of beetroot, R - native juice of beetroot

* - statistically significant differences for the proliferation value at the YS point and at the point for the juice after digestion and absorption in relation to the control

** - statistically significant differences for the proliferation value at point R and at the point for the juice after digestion and absorption in relation to the control

p≤0,05; the Mann-Whitney U test

(a)

(b)

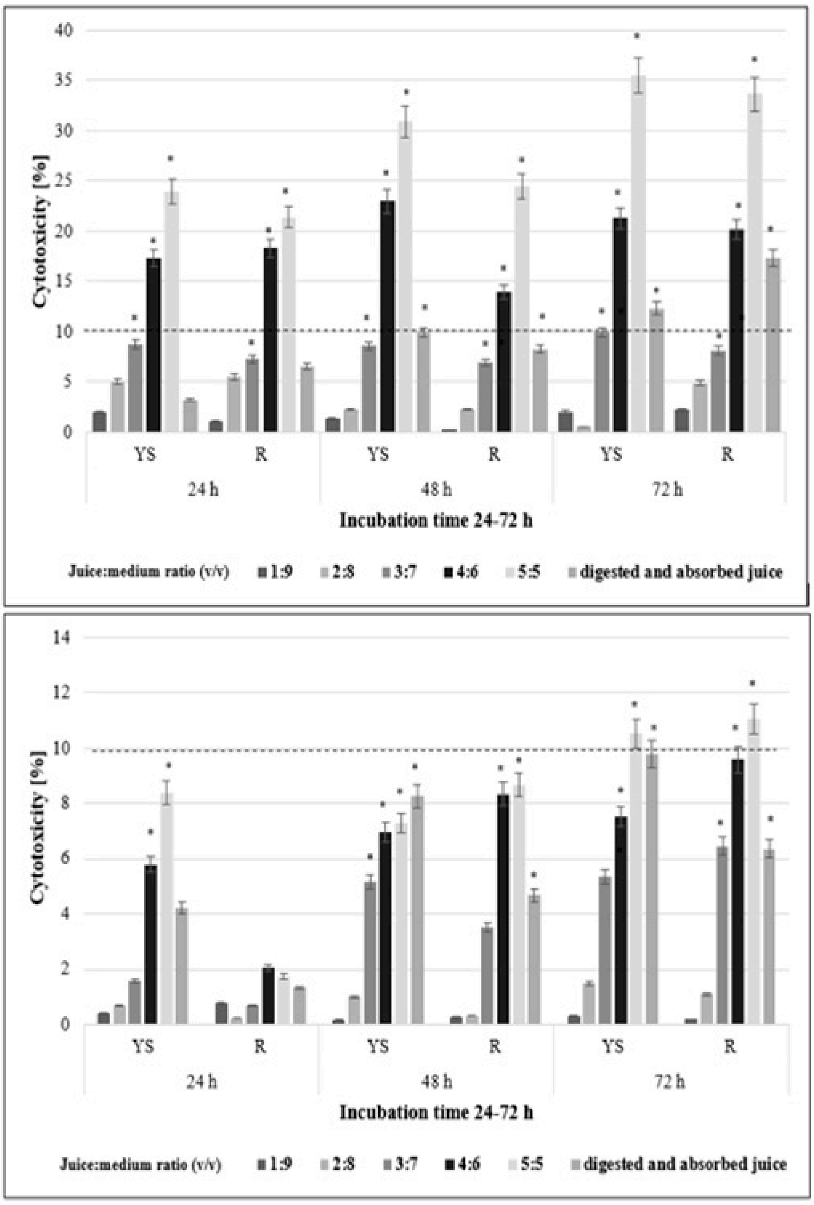

Supplementary Figure S3. Cytotoxicity of the tested material against MCF-7 (**a**) and MDA-MB-231 (**b**) cancer cells lines.

YS - native juice of young shoots of beetroot, R - native juice of beetroot

*$p \leqslant 0.05$ compared to controls; U Mann-Whitney test

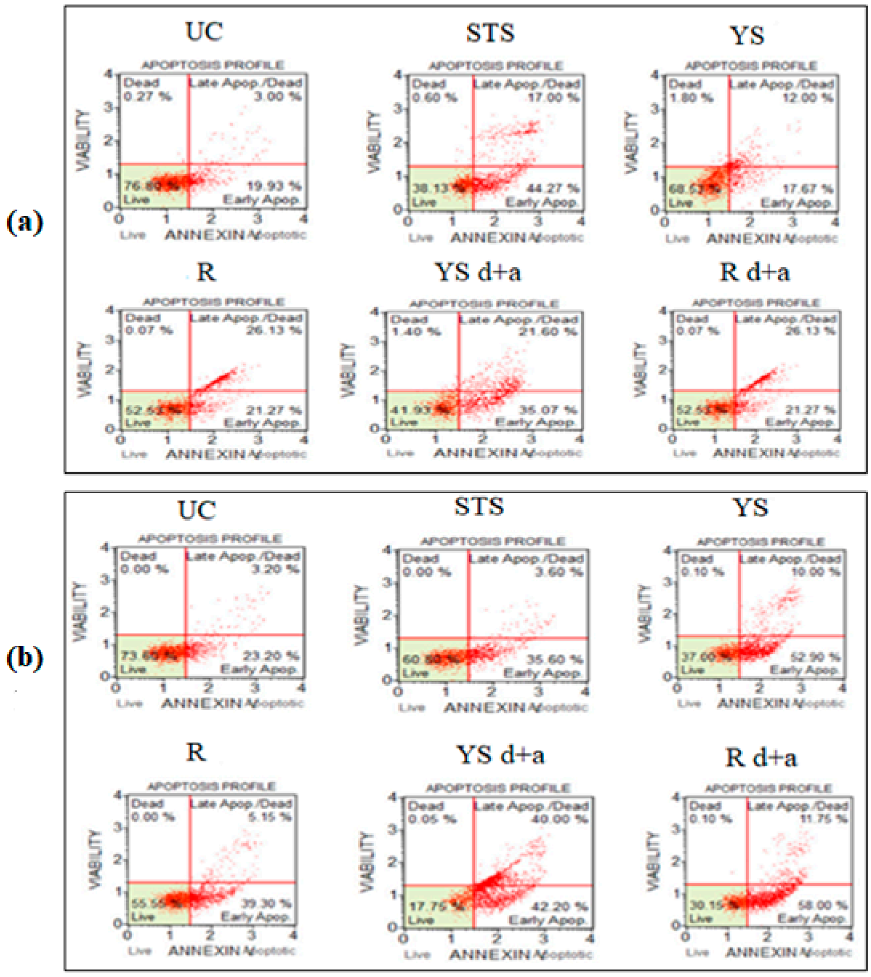

Supplementary Figure S4. The influence of the tested material and staurosporine on the induction of apoptosis in MCF-7 (**a**) and MDA-MB-231 (**b**) cancer cells lines.

UC - untreated control, STS - staurosporine, YS - native juice of young shoots of beetroot, R - native juice of beetroot, YS d+a - juice of young shoots of red beet subjected to *in vitro* gastrointestinal digestion and absorption, R d+a - juice of beetroot subjected to *in vitro* gastrointestinal digestion and absorption.

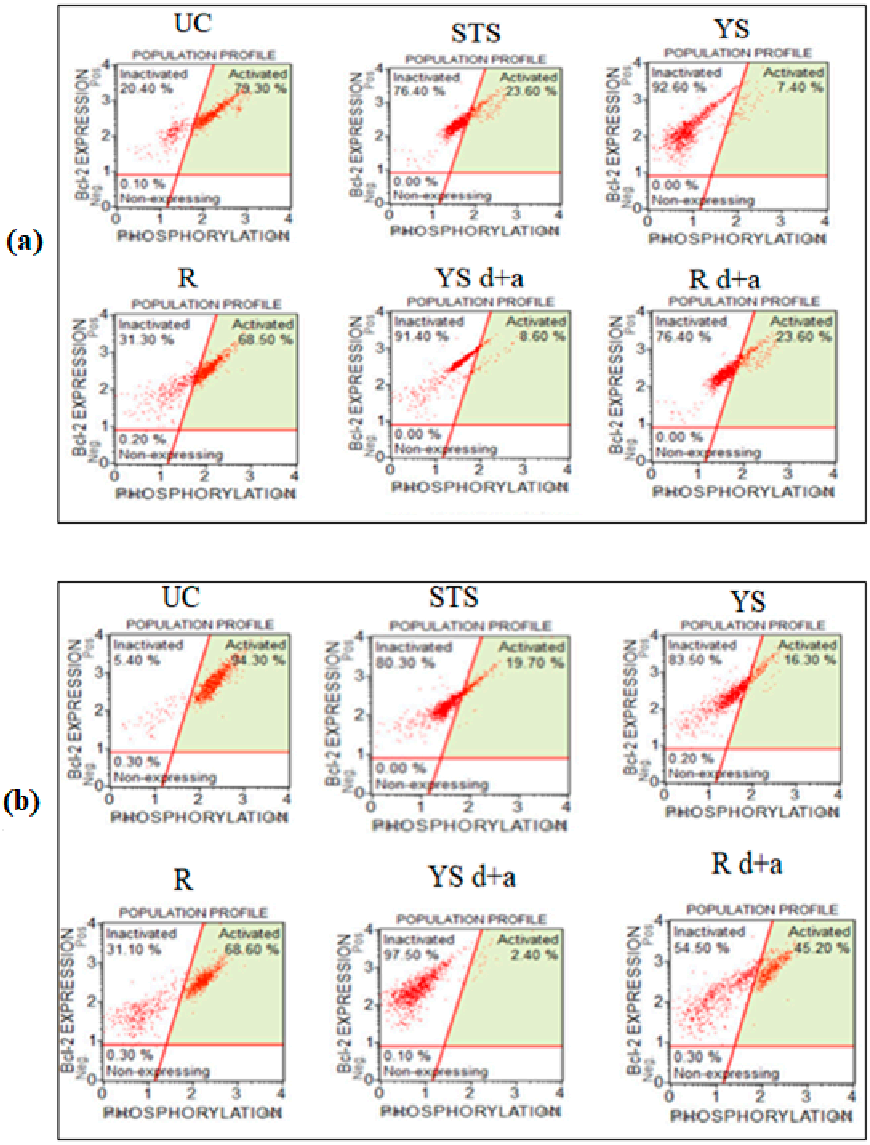

Supplementary Figure S5. The influence of the tested material and staurosporine on the inactivation of the BCL-2 protein in MCF-7 (**a**) and MDA-MB-231 (**b**) cancer cells lines.

UC - untreated control, STS - staurosporine, YS - native juice of young shoots of beetroot, R - native juice of beetroot, YS d+a - juice of young shoots of red beet subjected to *in vitro* gastrointestinal digestion and absorption, R d+a - juice of beetroot subjected to *in vitro* gastrointestinal digestion and absorption.

(a)

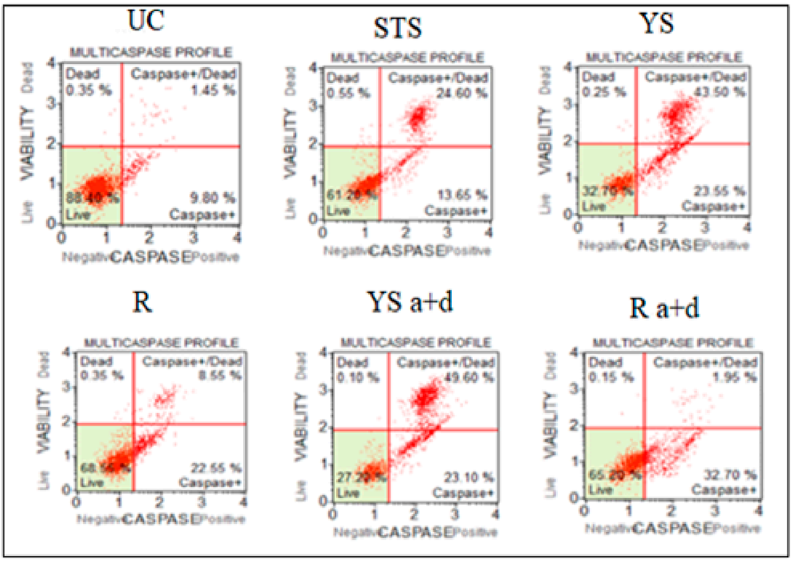

(b)

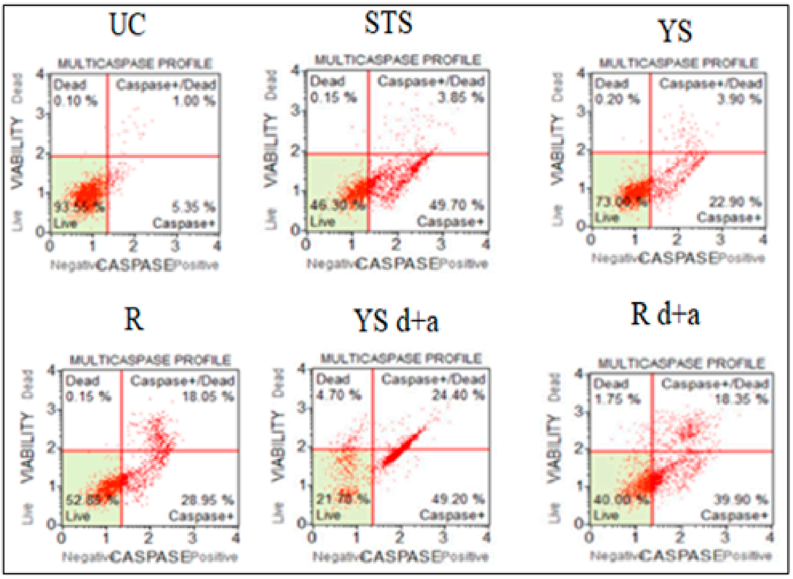

Supplementary Figure S6. The influence of the tested material and staurosporine on the activation of caspases in MCF-7 (**a**) and MDA-MB-231 (**b**) cancer cells lines.

UC - untreated control, STS - staurosporine, YS - native juice of young shoots of beetroot, R - native juice of beetroot, YS d+a - juice of young shoots of red beet subjected to *in vitro* gastrointestinal digestion and absorption, R d+a - juice of beetroot subjected to *in vitro* gastrointestinal digestion and absorption.

(a)

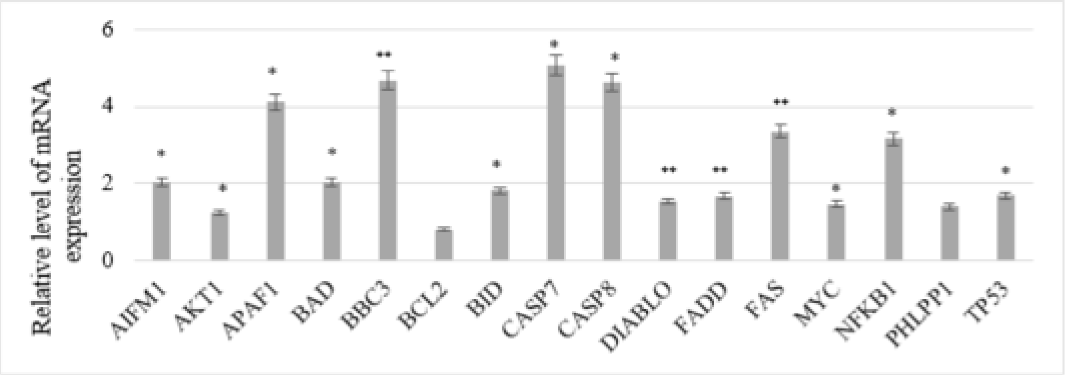

(b)

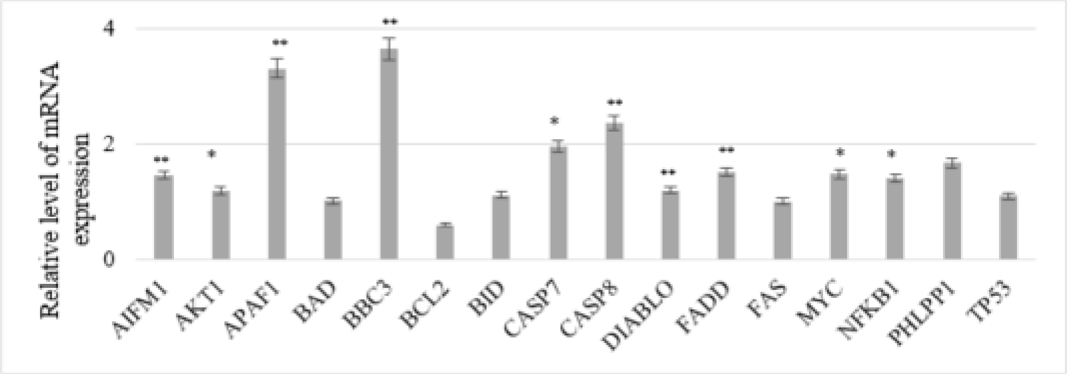

(c)

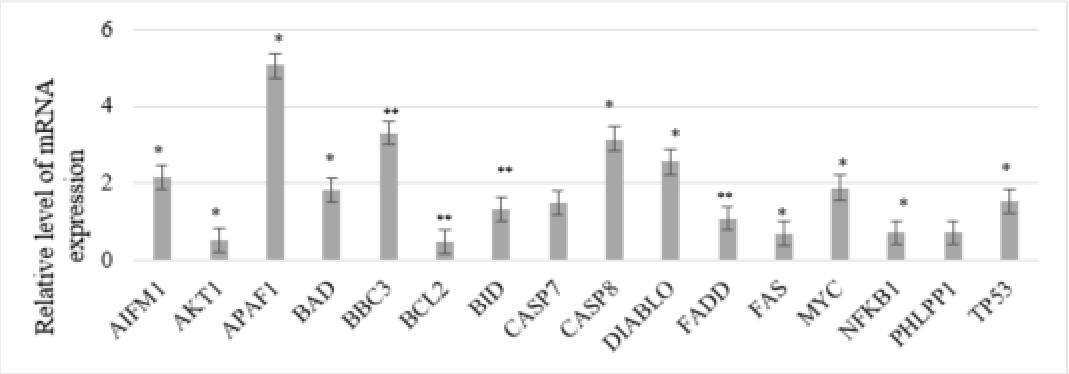

(d)

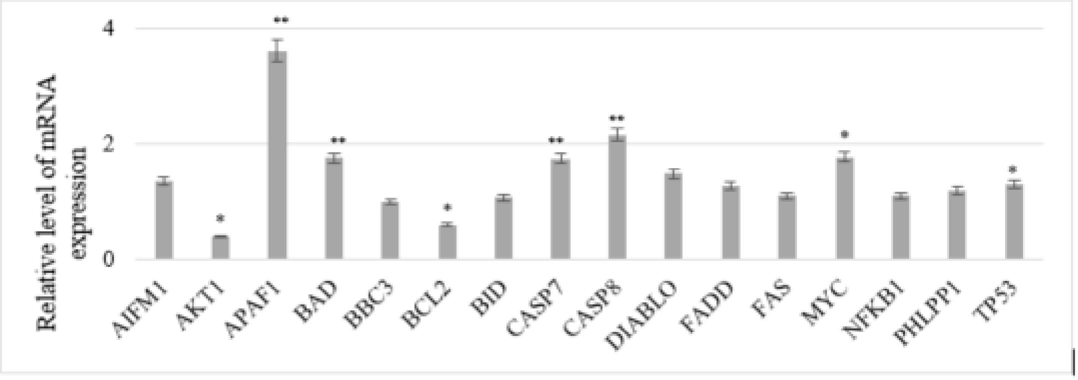

Supplementary Figure S7. Expression of selected genes associated with apoptosis and the cell cycle in MCF-7 cancer cell line, as a result of the action of YS (a), R (b), YS d+a (c), R d+a (d)

YS - native juice of young shoots of beetroot, R - native juice of beetroot, YS d+a - juice of young shoots of red beet subjected to *in vitro* gastrointestinal digestion and absorption, R d+a - juice of beetroot subjected to *in vitro* gastrointestinal digestion and absorption.

* $p \leq 0.05$ compared to controls; U Mann-Whitney test

** $p \leq 0.1$ compared to controls; U Mann-Whitney test

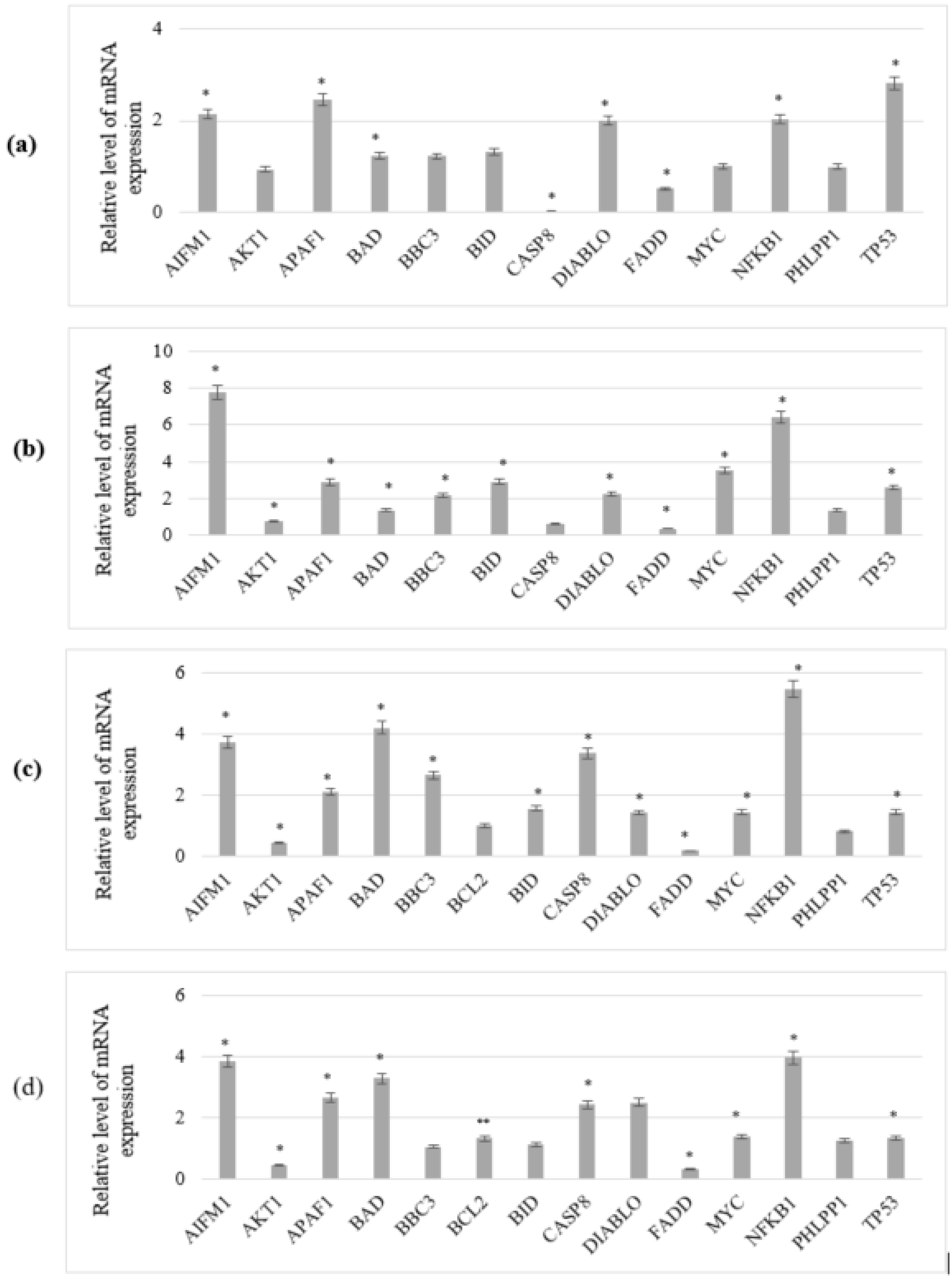

Supplementary Figure S8. Expression of selected genes associated with apoptosis and the cell cycle in MDA-MB-231 cancer cell line, as a result of the action of YS (a), R (b), YS d+a (c), R d+a (d)

YS - native juice of young shoots of beetroot, R - native juice of beetroot, YS d+a - juice of young shoots of red beet subjected to *in vitro* gastrointestinal digestion and absorption, R d+a - juice of beetroot subjected to *in vitro* gastrointestinal digestion and absorption.

* $p \leq 0.05$ compared to controls; U Mann-Whitney test

** $p \leq 0.1$ compared to controls; U Mann-Whitney test

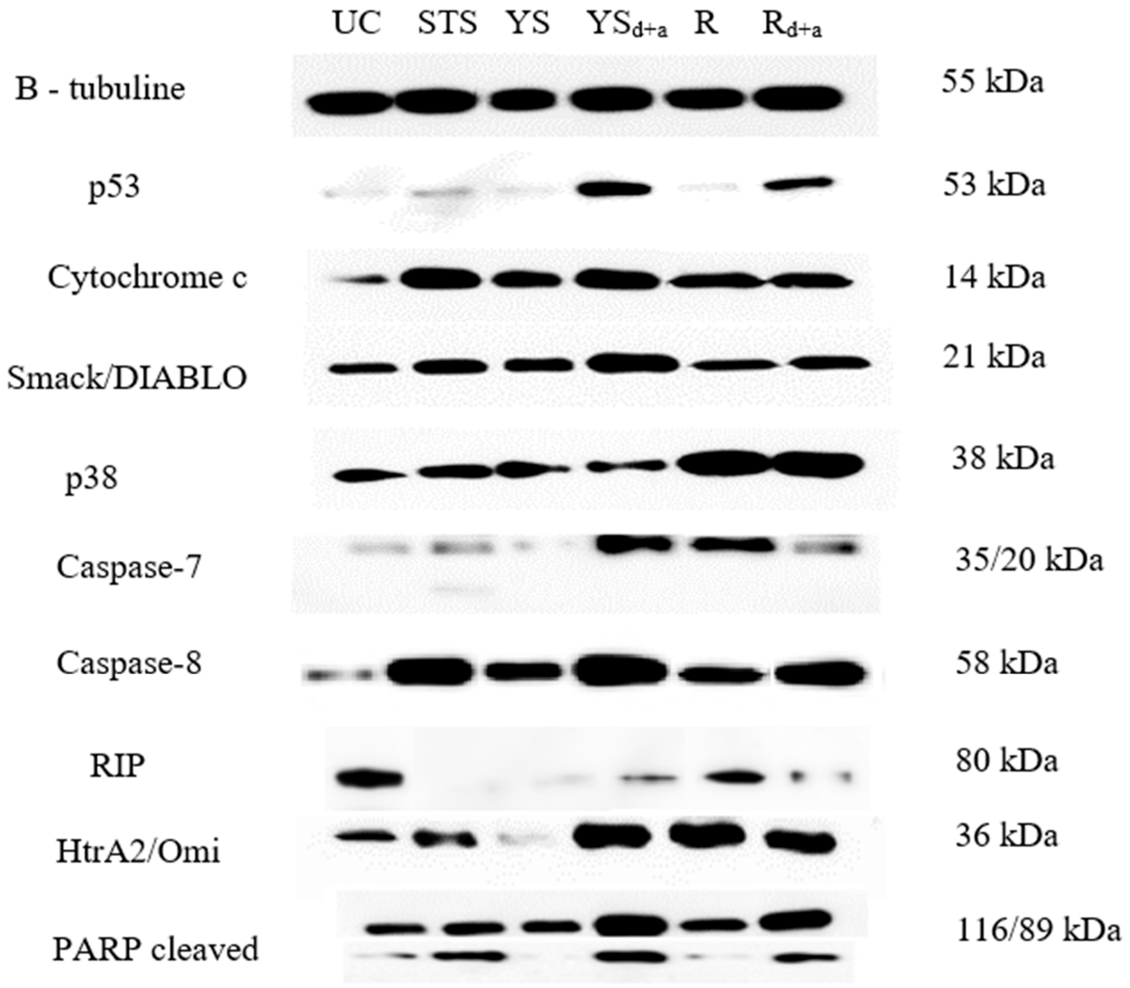

Supplementary Figure S9. Expression of selected apoptosis-related proteins in MCF-7 cancer cells line.

UC - untreated control, STS - staurosporine, YS - native juice of young shoots of beetroot, R - native juice of beetroot, YS d+a - juice of young shoots of red beet subjected to *in vitro* gastrointestinal digestion and absorption, R d+a - juice of beetroot subjected to *in vitro* gastrointestinal digestion and absorption,

p53

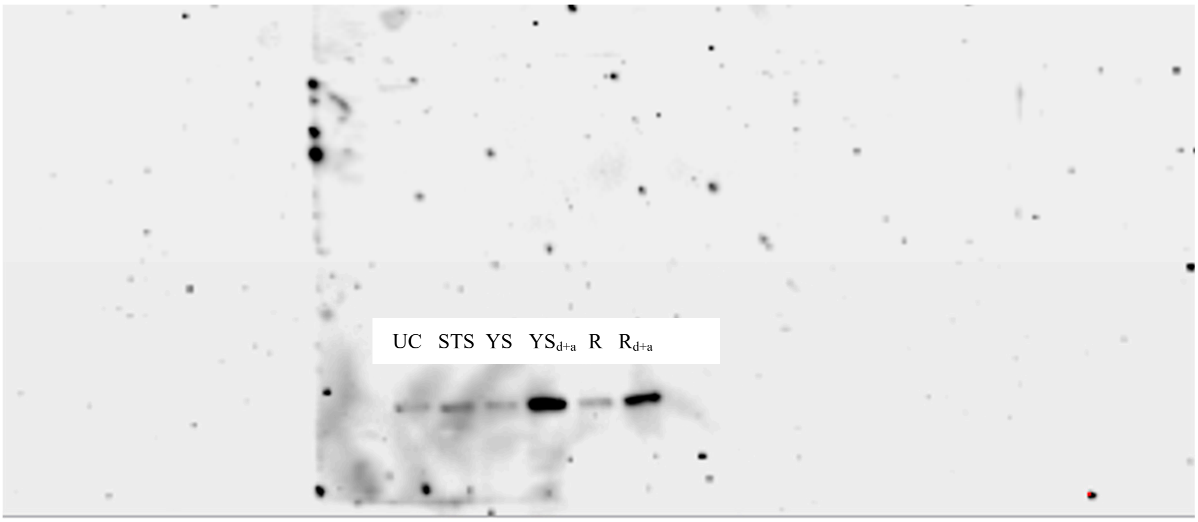

Cyt c

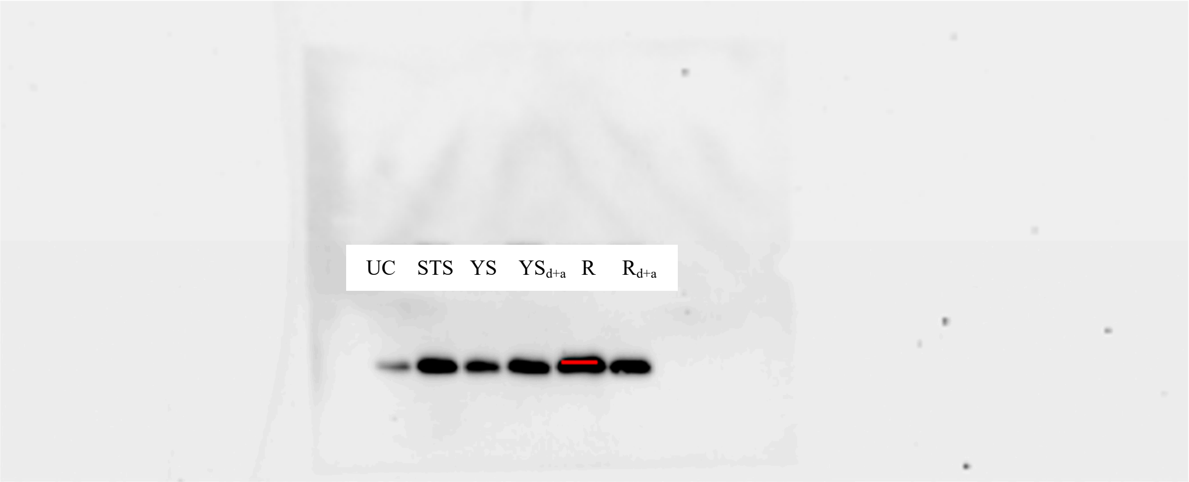

p38

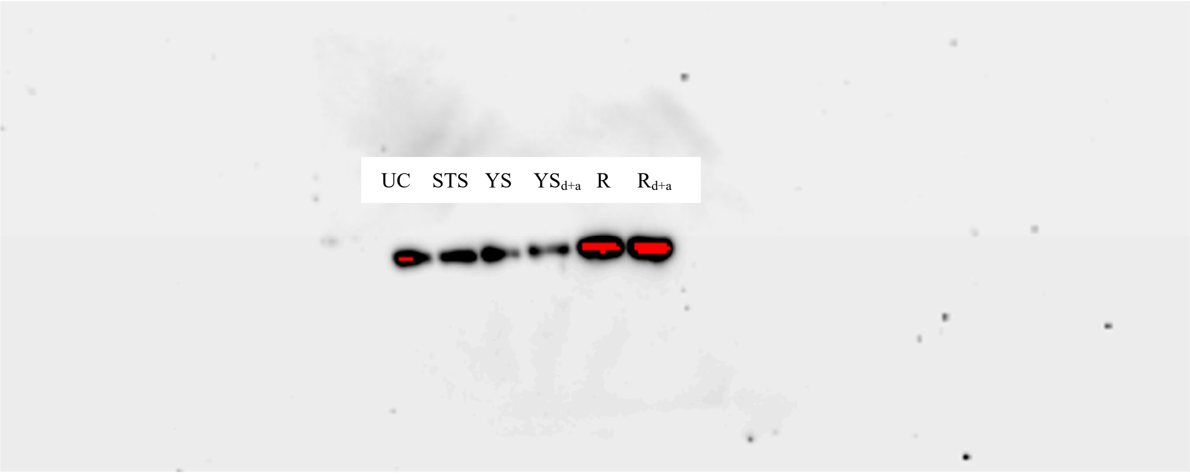

Caspase –7

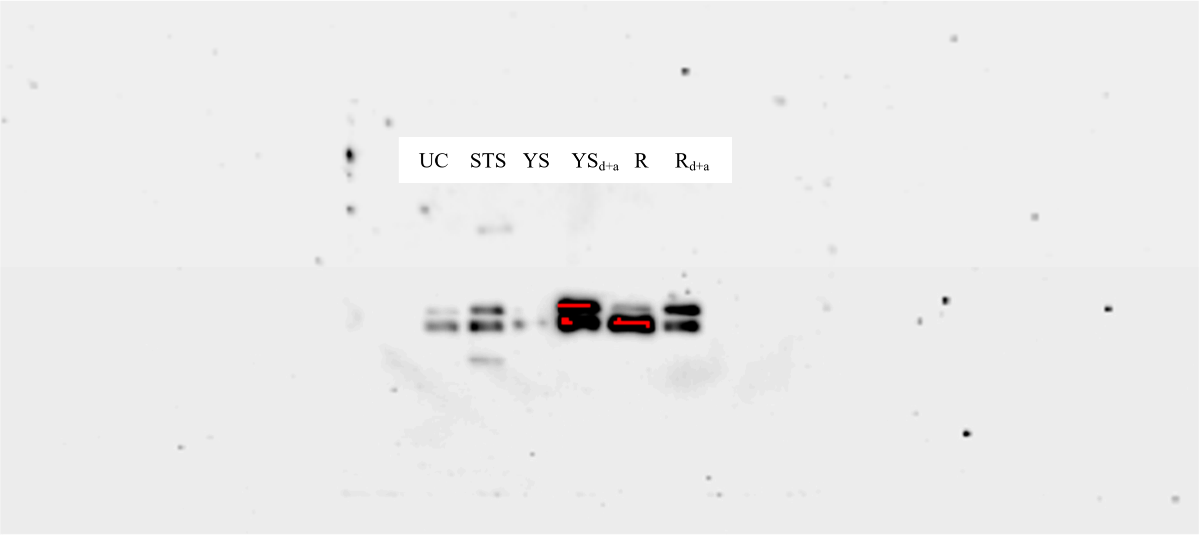

HtrA2/Omi

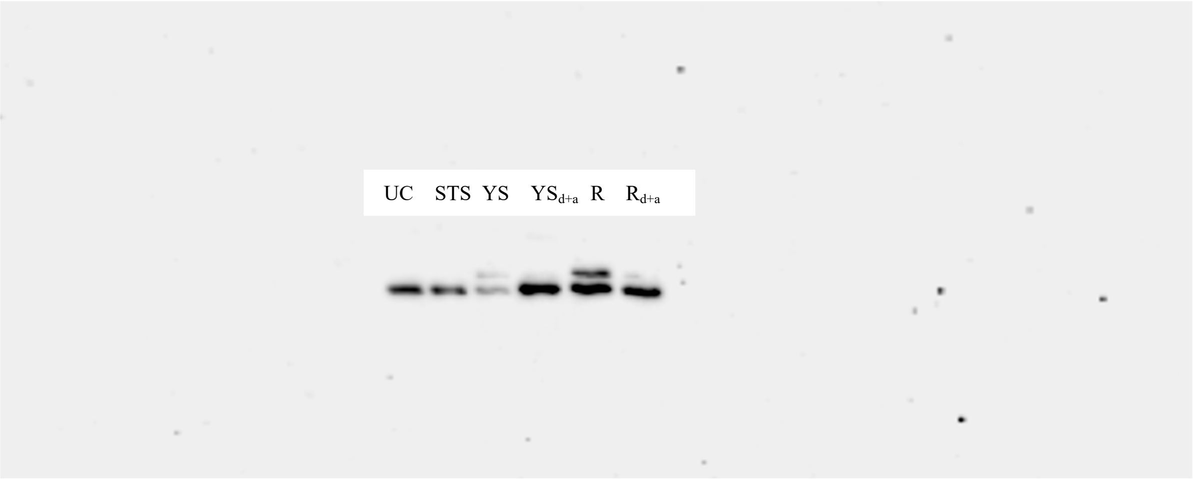

Supplementary Figure S10. Selected western blots images in MCF-7 cancer cells line.

UC - untreated control, STS - staurosporine, YS - native juice of young shoots of beetroot, R - native juice of beetroot, YS d+a - juice of young shoots of red beet subjected to *in vitro* gastrointestinal digestion and absorption, R d+a - juice of beetroot subjected to *in vitro* gastrointestinal digestion and absorption,

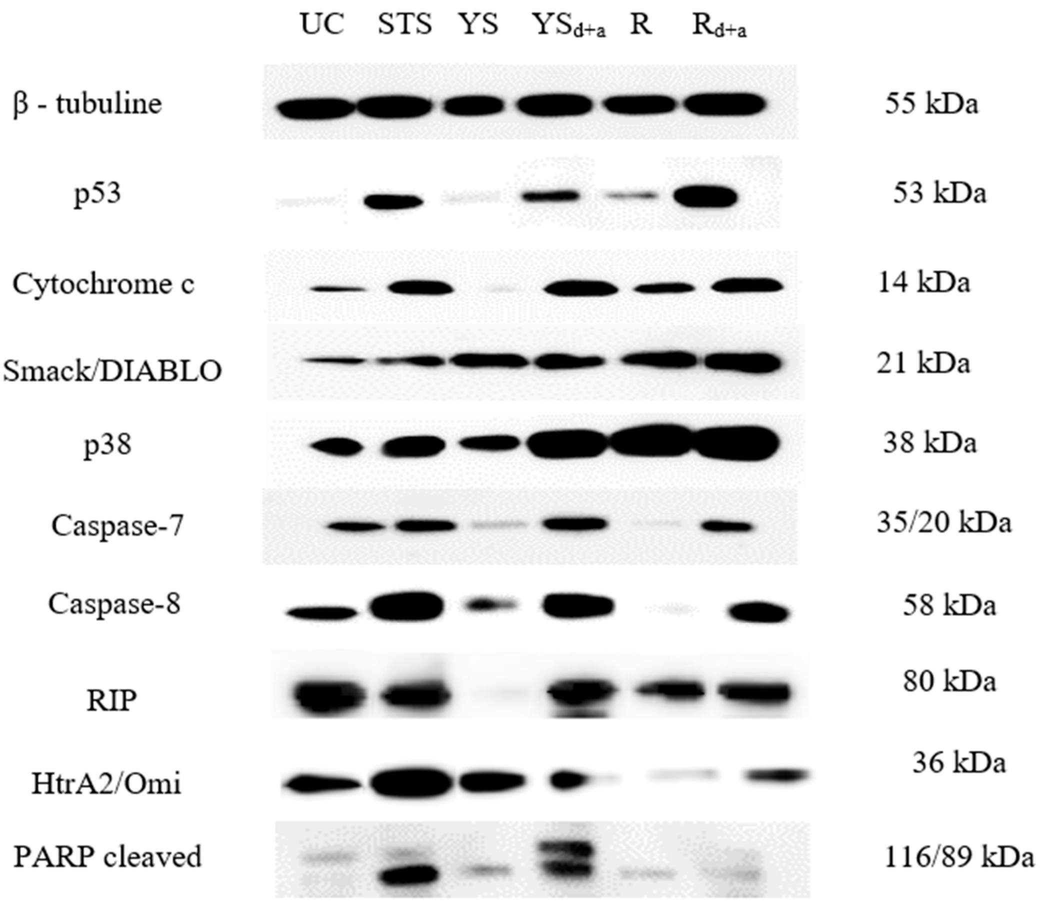

Supplementary Figure S11. Expression of selected apoptosis-related proteins in MDA-MB-231 cancer cells line.

UC - untreated control, STS - staurosporine, YS - native juice of young shoots of beetroot, R - native juice of beetroot, YS d+a - juice of young shoots of red beet subjected to *in vitro* gastrointestinal digestion and absorption, R d+a - juice of beetroot subjected to *in vitro* gastrointestinal digestion and absorption,

Cytochrome c

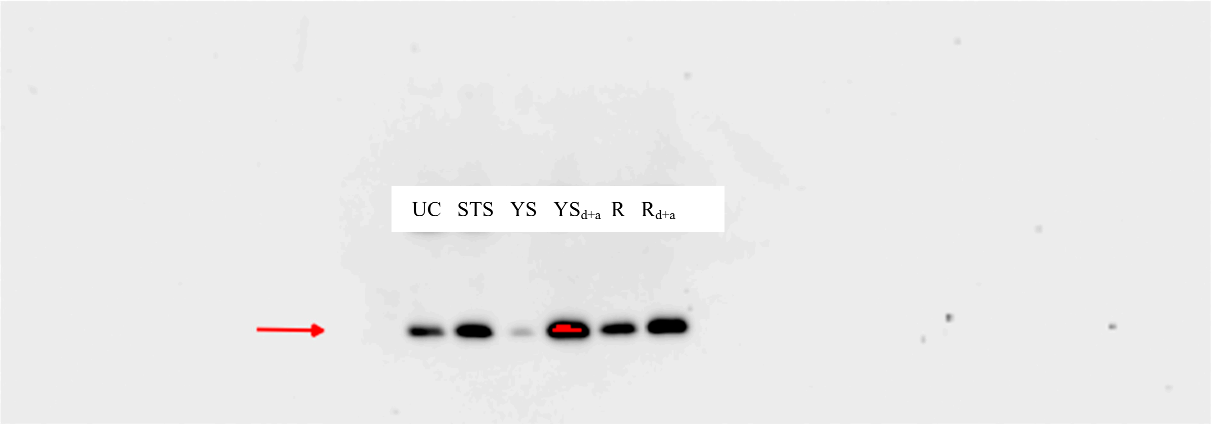

Smack/DIABLO

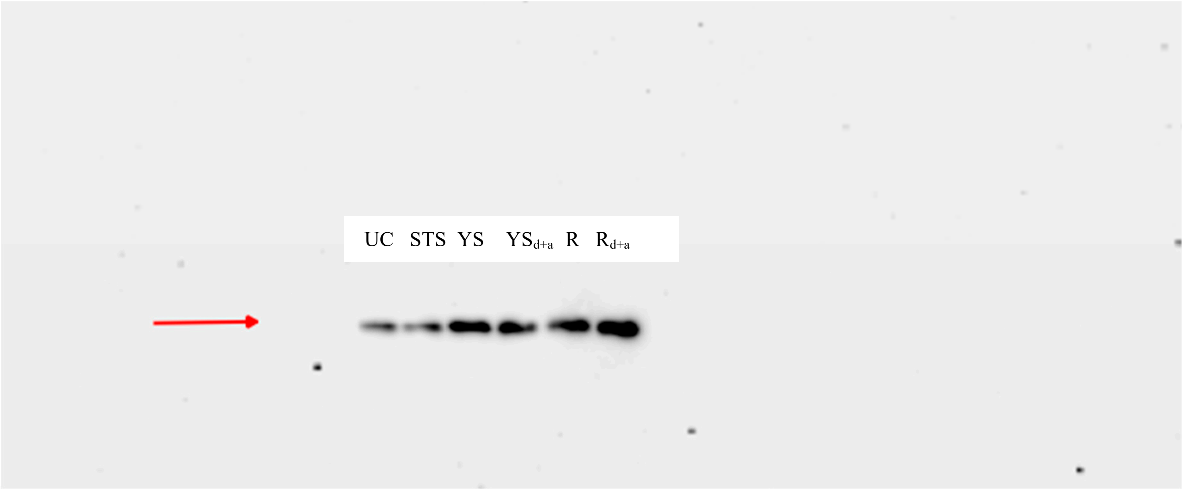

p38

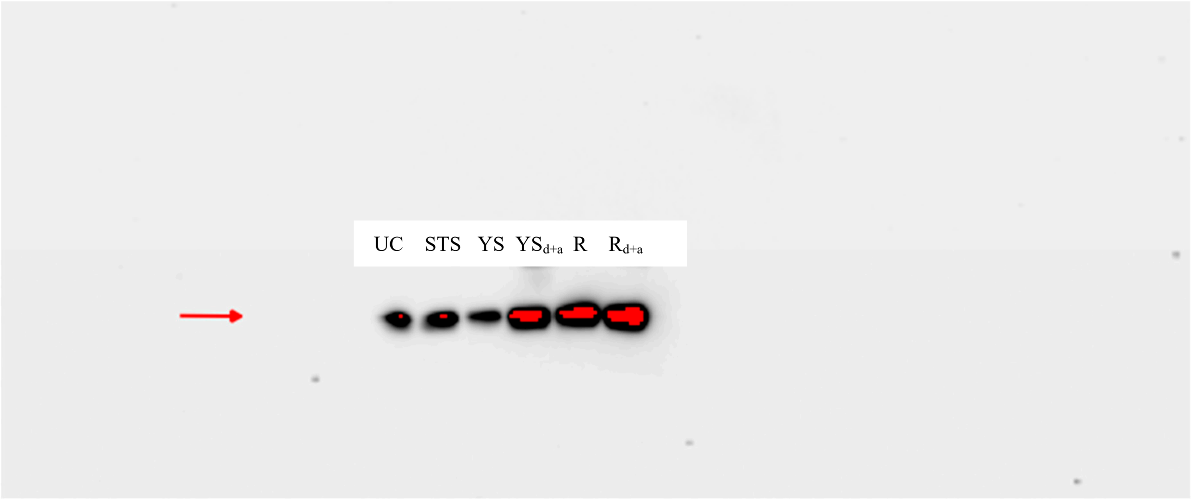

Caspase-7

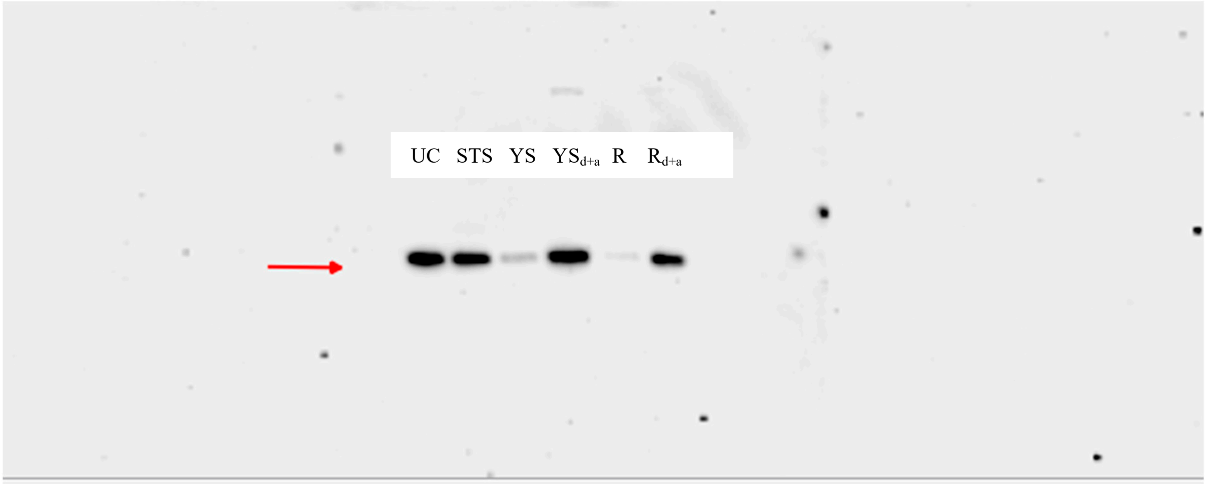

RIP

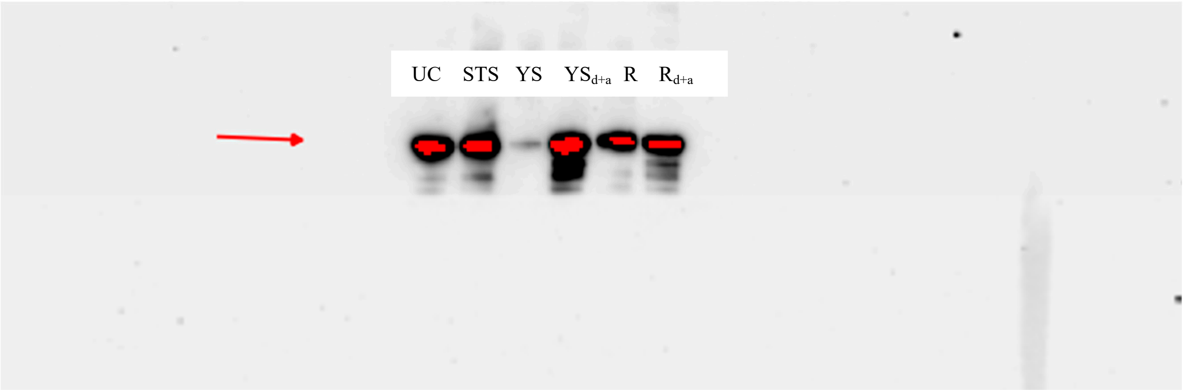

PARP cleaved

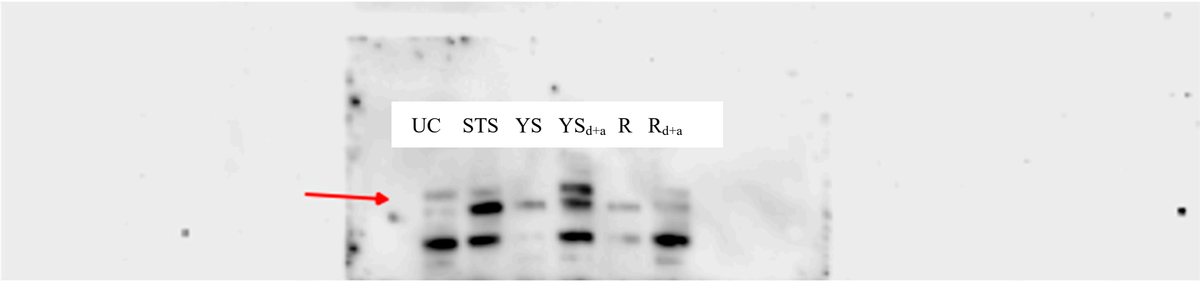

Supplementary Figure S12. Selected western blots images in MDA-MB-231 cancer cells line.

UC - untreated control, STS - staurosporine, YS - native juice of young shoots of beetroot, R - native juice of beetroot, YS d+a - juice of young shoots of red beet subjected to *in vitro* gastrointestinal digestion and absorption, R d+a - juice of beetroot subjected to *in vitro* gastrointestinal digestion and absorption,
